# Assessment of Chinese medicine for coronavirus-related pneumonia: A protocol for systematic review and meta-analysis

**DOI:** 10.1097/MD.0000000000020613

**Published:** 2020-06-12

**Authors:** Yibing Zhu, Zhiming Jiang, Yuke Zhang, Qi Zhang, Wen Li, Chao Ren, Renqi Yao, Jingzhi Feng, Yu Ren, Lin Jin, Yang Wang, Bin Du, Wei Li, Huibin Huang, Xiuming Xi

**Affiliations:** a Medical Research and Biometrics Center, Fuwai Hospital, National Center for Cardiovascular Diseases, Chinese Academy of Medical Sciences and Peking Union Medical College; b Department of Critical Care Medicine, Fuxing Hospital, Capital Medical University, Beijing; c Department of Respiratory Medicine, Qilu Hospital of Shandong University; d School of Medicine, Shandong University; e Department of Pulmonary and Critical Care Medicine, The First Affiliated Hospital of Shandong First Medical University; f Healthcare Big Data Institute, Shandong University, Jinan; g Trauma Research Center, Fourth Medical Center of the Chinese PLA General Hospital, Beijing; h Department of Burn Surgery, Changhai Hospital, the Second Military Medical University, Shanghai; i Department of Critical Care Medicine, Beijing Tsinghua Chang Gung Hospital; j Engineering Department, Google Inc.; k Medical ICU, Peking Union Medical College Hospital, Peking Union Medical College and Chinese Academy of Medical Sciences, Beijing, China.

**Keywords:** 2019 novel coronavirus disease, Chinese medicine, severe acute respiratory syndrome, systematic review

## Abstract

**Background:**

The 2019 novel coronavirus disease has caused a global pandemic with substantial morbidity and mortality. Chinese medicine has been extensively employed in the coronavirus-related pandemic in China. We aim to assess the efficacy and safety of Chinese medicine in treatment of coronavirus-related pneumonia with the updated results of relevant clinical trials.

**Methods:**

Six electronic databases including PubMed, EMBASE, Cochrane Library, China National Knowledge Infrastructure, Chongqing VIP, and SinoMed will be searched to identify randomized controlled trials up to May 2020. Patients diagnosed with coronavirus-related pneumonia including severe acute respiratory syndrome, Middle East respiratory syndrome, and 2019 novel coronavirus disease and administrated with Chinese medicine will be included. The primary outcome is the all cause mortality at the longest follow up available. The second outcomes include the length of stay in hospital and intensive care units, the duration of mechanical ventilation, and adverse events. The pooled effects will be analyzed and reported as risk ratios for dichotomous data using the Mantel–Haenszel method or mean differences for continuous data using the inverse-variance method. Sensitivity and subgroup analyses will be performed to test the robustness of the results and to explore the potential sources of heterogeneities. The Egger test and/or funnel plots will be used for the examination of publication bias. The grades of recommendation assessment, development, and evaluation methodology will be used to summarize the quality of evidence. The trial sequential analysis will be conducted to test whether the meta-analysis has a sufficient sample size after adjustment of the increased type I and II error risks.

**Results:**

The evidence to date of Chinese medicine in treatment of coronavirus-related pneumonia will be systematically reviewed and meta-analyzed.

**Conclusion:**

The relevant studies will be summarized and further evidence will be provided.

PROSPERO registration number: CRD42020178879

## 1 Introduction

The 2019 novel coronavirus disease (COVID-19) is a respiratory infection by a newly coronavirus and has been defined as a global pandemic by the World Health Organization.^[1]^ Once the patients develop severe respiratory failure and/or associated complications, they often require intensive care unit admission and are associated with substantial morbidity and mortality.^[2]^ This may due to no effective vaccines or specific treatments available currently on this new infection.^[3]^ Therefore, many potentially effective treatments have been put into clinical practice and trials.^[3,4]^ Among them, traditional Chinese medicine was the most frequently reported especially in China.^[5,6]^ Chinese medicine had been extensively employed as adjunctive treatment for the severe acute respiratory syndrome (SARS) epidemic in 2003.^[7,8]^ A meta-analysis had indicated that the adjunctive Chinese medicine treatment might be associated with improved symptoms and quality of life, reduction in corticosteroid use and hospitalization in SARS patients, as well as less mortality rate.^[8–10]^ However, suboptimal methodological quality of the included studies might affect the robustness of the conclusion. Furthermore, whether this advantage of Chinese medicine could also exhibit in COVID-19 remains unclear, though COVID-19 has the most similarity with SARS, followed by the Middle East respiratory syndrome (MERS).^[11,12]^ Recently, several articles focusing on Chinese medicine for COVID-19 has been published.^[6,13]^ Therefore we aimed to perform a systematic review and meta-analysis of the randomized controlled trials (RCTs) to evaluate the efficacy and safety of Chinese medicine in treatment of COVID-19. Furthermore, we would also assess whether treatment effects might vary among these coronavirus (ie, SARS, MERS, and COVID-19).

## 2 Review question

To assess the efficacy and safety of Chinese medicine in treatment of coronavirus-related pneumonia, including SARS, MERS, and COVID-19.

## 3 Methods

### 3.1 Study registration

This systematic review and meta-analysis was registered on the PROSPERO registration website (CRD42020178879) in accordance with the PRISMA-P guideline.^[14]^

### 3.2 Search methods

Six electronic databases (PubMed, EMBASE, Cochrane Library, the China National Knowledge Infrastructure, Chongqing VIP, and SinoMed) will be searched without language restriction to identify RCTs published from inception to May 2020. The reference lists of the relevant articles will also be searched. A search strategy has been developed using a combination of “coronavirus OR corona virus OR coronavirus-related OR SARS OR severe acute respiratory syndrome OR SARS-CoV MERS OR middle east respiratory syndrome OR MERS-CoV OR 2019nCoV OR COVID-19 OR SARS-CoV-2 OR novel coronavirus OR NCP” and “Chinese medicine OR traditional Chinese OR TCM or Chinese herb” in all fields. The searches will be re-run before the final analysis.

### 3.3 Inclusion criteria

#### 3.3.1 Studies

Only randomized control studies will be included.

#### 3.3.2 Participants

The study subjects consists of patients diagnosed with coronavirus-related pneumonia of any sex and age.

#### 3.3.3 Interventions/comparators

Chinese medicine, including extracts from herbs, as single or mixed formulas, regardless of their composition or form are administrated as the intervention for coronavirus-related pneumonia, in comparison with any other non-Chinese medicine pharmacological intervention, placebo, or no intervention.

#### 3.3.4 Outcomes

The primary outcome is the all cause mortality at the longest follow up available.

The second outcome measures include the length of stay in hospital, the length of stay in intensive care unit, the duration of mechanical ventilation, and adverse events.

### 3.4 Exclusion criteria

We exclude the studies available only in the abstract form or meeting reports.

### 3.5 Data collection and analysis

#### 3.5.1 Study screening

The identified records will be exported into EndNote X9 software to identify duplicates. After removal of the duplicates, the 3 reviewers (YBZ, ZMJ, and YKZ) will independently screen the title and abstract of the records. The full text of the potentially relevant articles will be obtained and the reference lists of which will also be screened. The selection process will be summarized and reported as a flow chart.

#### 3.5.2 Data extraction

The 2 reviewers (YBZ and ZMJ) will independently extract the publication information, study design, patient characteristics, interventions, and outcomes of each study using a predesigned extraction table. Any discrepancies will be discussed and resolved in discussion with a third reviewer (YKZ).

#### 3.5.3 Assessment of study quality

The 2 reviewers (YBZ and ZMJ) will independently assess the quality of the included studies using both of the Cochrane Collaboration's tool.^[15]^ The summary of each risk of bias item for each included study and each risk of bias item presented as percentages across all the included studies will be reported using a colored figure. The Modified Jadad Score will be applied for the assessment of exclusion criteria. The quality of evidence will be summarized and reported as the grades of recommendation assessment, development, and evaluation table.^[16,17]^ Any disagreements between the 2 reviewers will be solved by a consulting group including 2 experts (HBH and XMX).

#### 3.5.4 Statistical analyses and data synthesis

Review Manager 5.3 will be used for data synthesis. The synthesis of data requires for at least 4 RCTs. The studies not included in the quantitative synthesis will also be summarized and reported in the review. The pooled effects will be analyzed and reported as

(1) risk ratios for dichotomous data using the Mantel–Haenszel method; or
(2) mean differences for continuous data using the inverse-variance method; and
(3) 95% confidence intervals.

A 2-sided *P*-value of less than .05 is considered as statistical significance.

#### 3.5.5 Assessment of heterogeneity

The statistical heterogeneity will be detected by a standard Chi-square test with the *P*-value and *I*^*2*^ reported. The significance level of the *P*-value is .01 for this Chi-square test.^[18]^ The value of *I*^*2*^ suggests the level of heterogeneity (0%–40% insignificant, 30%–60% medium, 50%–90% substantial, 76%–100% high).^[18]^ The fixed effect model will be used if there is optimal homogeneity between the studies, including optimal clinical homogeneity and low statistical heterogeneity of *I*^2^ less than 30%.^[18]^ Otherwise, the random effect model will be chosen.

#### 3.5.6 Subgroup and sensitivity analyses

The subgroup analysis will be performed to explore the potential sources of heterogeneities and improve the clinical homogeneity in the subsets. The subgroups include

(1) different Chinese medicine;
(2) different comparators;
(3) different coronavirus (SARS, MERS, or COVID-19); and
(4) subjects of different age.

The sensitivity analysis will be conducted to test the robustness of the results by excluding each single RCT.

#### 3.5.7 Assessment of publication bias

The funnel plot will be used to detect the potential publication bias when at least 10 studies are included in the data synthesis.^[19]^ The Egger test will be conducted for studies less than 10.

#### 3.5.8 Trial sequential analysis (TSA)

The TSA will be performed to determine whether the meta-analysis has a sufficient sample size after adjustment of the increased type I and II error risks caused by multiple data merges.^[20]^ The Copenhagen TSA software will be used for the TSA analysis.

## 4 Discussion

Since Chinese medicine has accumulated experiences in the prevention and treatment of pandemic and endemic diseases for thousands of years, it is taken as the gem of the nation and the wealth of human medicine.^[13]^ Application of Chinese medicine as the adjunctive treatment of COVID-19 is largely inspired by the experience in the SARS management.^[13,21]^ The previous studies indicated associations of Chinese medicine and improved symptoms in SARS.^[8–10]^ Without specific antiviral agents in COVID-19 yet, the foremost way of treatment remains symptomatic. First, to improve symptoms is one of the advantages of Chinese medicine.^[8–10]^ Furthermore, Chinese medicine also showed associations with other improved clinical outcomes in SARS, including reduction in corticosteroid use, length of stay in hospital, and mortality rate.^[8–10]^ In the battle against COVID-19, Chinese medicine has also been highly valued and widely used. Since the efficacy and safety of Chinese medicine in treatment of COVI-D19 are needed to be proved, many ongoing studies (50 trials registered up to March 1, 2020) in China are focused on this issue.^[21]^ Thus this systematic review and meta-analysis will summarize the studies and try to provide further evidence with the updated results.

## Author contributions

**Conceptualization:** Yibing Zhu, Huibin Huang, Xiuming Xi.

**Data curation:** Yibing Zhu, Zhiming Jiang, Yuke Zhang.

**Methodology:** Yang Wang, Xiuming Xi, Bin Du.

**Project administration:** Yibing Zhu.

**Software:** Huibin Huang, Chao Ren, Renqi Yao.

**Supervision:** Wei Li, Lin Jin.

**Writing – original draft:** Yibing Zhu.

**Writing – review and editing:** Wen Li, Jingzhi Feng, Yu Ren, Huibin Huang, Qi Zhang.
